# Modulation of immune and inflammatory responses involving selected galectins and selected immune-inflammatory mediators in women with deep endometriosis coexisting with adenomyosis – preliminary study

**DOI:** 10.3389/fendo.2026.1920315

**Published:** 2026-10-13

**Authors:** Dominika Wendlocha, Marta Smycz-Kubańska, Magdalena Kościelniak-Ziemniak, Mariusz Wójtowicz, Aleksandra Mielczarek-Palacz

**Affiliations:** 1 Department of Immunology and Serology, Faculty of Pharmaceutical Sciences in Sosnowiec, Medical University of Silesia in Katowice, Katowice, Poland; 2 Medical Diagnostic Laboratory City Hospital in Zabrze, Zabrze, Poland; 3 Clinical Department of Gynecology and Obstetrics, Faculty of Medical Sciences in Zabrze, Medical University of Silesia in Katowice, Katowice, Poland

**Keywords:** deep infiltrating endometriosis, adenomyosis, galectins, inflammation, immune response

## Abstract

**Introduction:**

Endometriosis is characterized by the presence of functionally active endometrial tissue outside the uterine cavity, primarily within the peritoneal cavity. Its etiology and pathogenesis remain incompletely understood; however, endometriosis is considered a chronic inflammatory disease associated with immune system dysfunction. Galectins and other mediators of immune and inflammatory responses may play an important role in the pathogenesis of the disease. This study aimed to investigate the potential involvement of selected immune and inflammatory mediators in endometriosis by assessing the concentrations of galectin-1 (Gal-1), galectin-3 (Gal-3), galectin-8 (Gal-8), galectin-9 (Gal-9), asprosin, FSTL1, meteorin, neuregulin-4, isthmin 1, SFRP2, and TNF-α in the serum and peritoneal fluid of women with deep infiltrating endometriosis, deep infiltrating endometriosis coexisting with adenomyosis, and adenomyosis.

**Methods:**

The study included 33 women in whom endometriosis was diagnosed during laparoscopy and subsequently confirmed by histopathological examination. The reference group consisted of 14 women without endometriosis. Blood serum and peritoneal fluid samples were collected, and the concentrations of the investigated parameters were determined using enzyme-linked immunosorbent assays (ELISAs).

**Results:**

In patients with deep infiltrating endometriosis coexisting with adenomyosis, alterations in the detectability of Gal-1, Gal-3, and Gal-9 were observed, suggesting a potential involvement of these galectins in the regulation of immune and inflammatory responses. Differences in the concentrations of asprosin, FSTL1, and TNF-α were also observed in this group, suggesting a possible association of these parameters with the inflammatory environment accompanying the disease.

**Conclusions:**

The observed alterations in Gal-1, Gal-3, and Gal-9 levels in patients with deep infiltrating endometriosis coexisting with adenomyosis may indicate their involvement in the regulation of immune and inflammatory responses. Differences in the concentrations of asprosin, FSTL1, and TNF-α suggest their potential association with the inflammatory environment of the disease. Further studies in larger cohorts are needed to determine the biological and clinical significance of these parameters and their potential interactions.

## 1 Introduction

Endometriosis (EM) is an estrogen-dependent inflammatory gynecological disease characterized by the presence of endometrial tissue outside the uterine cavity. It presents in three main phenotypes: superficial peritoneal lesions, ovarian endometriomas, and deep endometriosis (DE), which often coexists with adenomyosis despite being a distinct condition. Endometriosis affects approximately 10% of women of reproductive age worldwide; however, its exact prevalence remains uncertain, and diagnosis is often delayed by 8–10 years (1). A major clinical concern is endometriosis-associated infertility, affecting 40–60% of patients (2).

Despite numerous studies and proposed theories, the pathogenesis of endometriosis remains unclear and is often referred to as the “disease of a thousand hypotheses”. Its development is influenced by genetic, environmental, and hormonal factors, as well as immune system dysregulation accompanied by chronic inflammation (3–6).

Peritoneal immune dysfunction leads to an impaired response against endometrial cells, promoting their survival, implantation, and development into endometriotic lesions. Key mediators of these immune and inflammatory processes, including galectins, play an important role in disease progression (7).

Galectins are glycan-binding proteins with affinity for β-galactosides, present both intracellularly and extracellularly, and involved in the regulation of cell–cell interactions, apoptosis, adhesion, migration, angiogenesis, invasion, and inflammation. Among human galectins, Gal-1, Gal-3, Gal-8, and Gal-9 are considered most relevant in the context of endometriosis and may contribute to ectopic endometrial growth. However, available data on their role in disease pathogenesis remain limited and inconclusive.

Increased galectin-1 expression has been reported in endometriotic lesions, while elevated serum levels have been associated with disease severity, angiogenesis, and local immunomodulation (8, 9). Similarly, galectin-3 has been implicated in lesion development through effects on cell proliferation and angiogenesis, and experimental studies suggest that its inhibition may reduce lesion growth (10–12). In contrast, data on galectin-8 in endometriosis are scarce, although its known involvement in cell adhesion and immune regulation suggests a potential role in lesion formation (13). Galectin-9 has been reported at elevated serum levels in patients with endometriosis, indicating potential diagnostic value as a non-invasive biomarker, although further validation is required (14).

The complex and not fully understood role of galectins in endometriosis prompted a broader analysis of their serum and peritoneal fluid concentrations in patients with different disease phenotypes. In addition to galectins, selected immune and inflammatory mediators, including asprosin, follistatin-like protein 1 (FSTL1), meteorin, neuregulin-4, isthmin-1 (ISM1), secreted frizzled-related protein 2 (SFRP2), and tumor necrosis factor-α (TNF-α), were evaluated as potentially involved in the regulation of inflammatory and metabolic processes in endometriosis (15–23). Most of these mediators have been only sparsely investigated in endometriosis, and asprosin has not been previously studied in this context. Available evidence suggests that asprosin is associated with metabolic regulation and may be linked to reproductive function and ovarian physiology (15). FSTL1 has been implicated in inflammatory responses and tissue remodeling processes, including angiogenesis and stromal activation, suggesting a potential role in lesion development (16, 17). Meteorin, neuregulin-4, ISM1, and SFRP2 have been associated with angiogenesis, metabolic signaling, immune regulation, and cell proliferation in various pathological conditions, although their role in endometriosis remains largely unexplored (18–22). TNF-α is a well-established proinflammatory cytokine involved in immune activation, cell adhesion, angiogenesis, and tissue remodeling, and its role in endometriosis has been widely described (23–25).

The aim of the study was to evaluate the concentrations of galectin-1, -3, -8, and -9, as well as asprosin, FSTL1, meteorin, neuregulin-4, ISM1, SFRP2, and TNF-α in serum and peritoneal fluid of women with deep endometriosis, deep endometriosis coexisting with adenomyosis, and adenomyosis.

## 2 Materials and methods

### 2.1 Study and control groups

The study group consisted of 33 Caucasian patients who underwent surgery for endometriosis at the Clinical Department of Gynecology and Obstetrics, Faculty of Medical Sciences in Zabrze, between 2023 and 2025.

The patients were divided into three clinical groups and control (**Table 1**).

**Table 1:** Characteristics of the study and control groups.

Group	Number of cases (N)	Age (me; Q1/Q3)	BMI (me; Q1/Q3)
Deep endometriosis (DE)	9	36 (34-37)	23.62 (19.27-24.61)
Coexisting adenomyosis and deep endometriosis	17	38 (29-42)	22.31(21.97-27.25)
Adenomyosis	7	43.5 (32-53)	23.3 (20.52- 26.03)
Control	14	47.5 (36-53)	25.95(23.15-26.7)

Exclusion criteria included thyroid dysfunction, Cushing’s syndrome, type 1 or type 2 diabetes, tobacco smoking, alcohol abuse, changes in body weight within the last three months, and any ongoing pharmacological treatment. The study was conducted after obtaining informed consent from each participant, in accordance with the study protocol approved by the Bioethics Committee of the Medical University of Silesia in Katowice (BNW/NWN/0052/KB/134/23, BNW/NWN/0052/KB1/111/24).

The control group consisted of 14 patients, selected based on the exclusion criteria. Exclusion criteria included suspected endometriosis based on clinical symptoms or imaging studies, chronic pelvic pain syndrome use of any hormonal therapy (including oral contraceptives, progestogens, GnRH analogs, or other hormonal agents) within the past three months, pregnancy or breastfeeding, and chronic or autoimmune diseases that could affect study outcomes. The control participants did not undergo laparoscopy specifically for the exclusion of endometriosis or adenomyosis; therefore, the absence of these conditions was based on clinical assessment and available imaging findings rather than surgical confirmation.

The study design and data collection were performed in accordance with the WERF EPHect recommendations where applicable.

### 2.2 Preparation of serum and peritoneal fluid

Blood samples were collected from patients in the morning from the antecubital vein into tubes containing a clot activator for serum and into EDTA tubes for whole blood. Complete blood counts and biochemical analyses were performed immediately after collection. The samples collected in clot activator tubes were allowed to clot for 30 minutes and then centrifuged at 1500 × g for 15 minutes at room temperature. The obtained serum was aliquoted and stored at −80 °C until analysis.

Peritoneal fluid was collected laparoscopically from the pouch of Douglas as free fluid present within the peritoneal cavity prior to the procedure, following the manufacturer’s kit instructions. The samples were then frozen and stored at −80 °C until analysis.

### 2.3 ELISA tests

The concentrations of the studied parameters in serum and peritoneal fluid were measured using an enzyme-linked immunosorbent assay (ELISA). All ELISA measurements were performed in a single batch under identical laboratory conditions to minimize inter-assay variability (**Table 2**).

**Table 2:** Tests performed and their specifications.

Parameter	Test name	Producer	Test method	Detection range	Sensitivity	Precision	
						Intra-assay	Inter-assay
Galectin-1	ELISA Kit For Galectin 1 (GAL1)	Cloud-Clone Corp.	Double-antibody Sandwich	0.312-20ng/mL.	<0.123ng/mL.	CV<10%	CV<12%
Galectin-3	ELISA Kit For Galectin 3 (GAL3) (SEA303Hu)	Cloud-Clone Corp.	Double-antibody Sandwich	0.156-10ng/ml.	<0.054ng/mL.	CV<10%	CV<12%
Galectin-8	ELISA Kit For Galectin 8 (GAL8) (SEA308Hu)	Cloud-Clone Corp.	Double-antibody Sandwich	0.156-10ng/mL	<0.063ng/mL	CV<10%	CV<12%
Galectin-9	ELISA Kit For Galectin 9 (GAL9)	Cloud-Clone Corp.	Double-antibody Sandwich	78-5,000pg/mL	< 33pg/mL	CV<10%	CV<12%
FSTL1	ELISA Kit for Follistatin Like Protein 1 (FSTL1) (SEJ085Hu)	Cloud-Clone Corp.	Double-antibody Sandwich	1.56–100 ng/mL	<0.59 ng/mL	CV<10%	CV<12%
Asprosin	ELISA Kit for Asprosin (Asp) (SEA332Hu)	Cloud-Clone Corp.	Double-antibody Sandwich	0.156–10 ng/mL	<0.064 ng/mL	CV<10%	CV<12%
Meteorin	ELISA Kit for Meteorin (METRN) (SEH662Hu)	Cloud-Clone Corp.	Double-antibody Sandwich	0.156–10 ng/mL	<0.057 ng/mL	CV<10%	CV<12%
Neuregulin 4	ELISA Kit for Neuregulin (NRG4) (SEC174Hu)	Cloud-Clone Corp.	Double-antibody Sandwich	0.156–10 ng/mL	<0.056 ng/mL	CV<10%	CV<12%
Secreted Frizzled Related Protein 2	ELISA Kit for Secreted Frizzled Related Protein 2 (SFRP2) (SEF879Hu)	Cloud-Clone Corp.	Double-antibody Sandwich	78-5.000pg/mL.	< 31pg/mL	CV<10%	CV<12%
Isthmin 1	ELISA Kit for Isthmin 1 (ISM1) (SEQ515Hu)	Cloud-Clone Corp.	Double-antibody Sandwich	0.156–10 ng/mL	<0.064 ng/mL	CV<10%	CV<12%
TNF α	Human TNF alpha ELISA Kit (950.090.096)	Diaclone	Double-antibody Sandwich	25–800 pg/ml	<8 pg/ml	CV<10%	CV<12%

### 2.4 Morphological and biochemical parameters

White blood cell count (WBC), platelet count (PLT), and hemoglobin concentration (HGB) were measured in whole blood samples using a SYSMEX XN 550 hematology analyzer.

C-reactive protein (CRP) and glucose (GLU) concentrations were determined using a Roche Integra biochemical analyzer, following the manufacturer’s instructions and standard laboratory procedures.

### 2.5 Statistical analysis

Statistical analysis was performed using Statistica software (StatSoft, USA). Continuous variables were expressed as median and interquartile range (Me, Q1–Q3).

For comparisons between groups, the Kruskal–Wallis test was used. *Post hoc* pairwise comparisons following the Kruskal–Wallis test were performed using Dunn’s test. Pairwise comparisons of related samples were performed using the Wilcoxon signed-rank test where appropriate. Associations between categorical variables were analyzed using Fisher’s exact test, while McNemar’s test was applied for paired categorical data.

Statistical analyses and selection of appropriate tests were additionally supported using available statistical calculators. A p-value < 0.05 was considered statistically significant.

Values exceeding the upper limit of quantification (ULOQ) were assigned the maximum measurable concentration of the assay (800 pg/mL) for statistical analysis. For measurements below the lower limit of quantification (LLOQ), the values were reported by the assay as below the detection limit. Due to the high proportion of measurements below the LLOQ for the galectins, quantitative analysis of these biomarkers was not considered appropriate. Therefore, galectin concentrations were analyzed as categorical variables based on detectability (detectable vs. non-detectable). This approach was applied consistently across all galectins. The potential influence of censored measurements and values exceeding the ULOQ on the statistical results was considered when interpreting the findings. For FSTL1, serum concentrations below the lower limit of quantification (LLOQ) were excluded from the quantitative analysis. Statistical analyses were therefore performed using only quantifiable FSTL1 concentrations.

## 3 Results

### 3.1 Galectins

The presence of galectin-1, -3, -8, and -9 in serum and peritoneal fluid was determined in patients with deep endometriosis coexisting with adenomyosis, deep endometriosis, adenomyosis, and controls.

Results were obtained for galectin-1, -3, and -9 as shown in the **Table 3** and **Figure 1**.

**Table 3:** Frequency of detectable galectin-1 (Gal-1), galectin-3 (Gal-3), and galectin-9 (Gal-9) results in the serum of patients with coexisting adenomyosis and DE, deep endometriosis (DE), and adenomyosis compared with the control group.

Marker	Group	Detectable n/N (%)	Control n/N (%)	p (Fisher’s test)/Bonferroni-adjusted p
Gal-1	Adenomyosis + DE	0/17 (0%)	8/14 (57.1%)	0.0004 */0.0036
	DE	2/9 (22.2%)	8/14 (57.1%)	0.1968
	Adenomyosis	1/7 (14.2%)	8/14 (57.1%)	0.1588
Gal-3	Adenomyosis + DE	6/17 (35.2%)	6/14 (42.8%)	0.7241
	DE	5/9 (55.5%)	6/14 (42.8%)	0.4003
	Adenomyosis	2/7 (28.5%)	6/14 (42.8%)	0.6494
Gal-9	Adenomyosis + DE	15/17 (88.2%)	4/14 (28.5%)	0.0011*/0.0099
	DE	4/9 (44%)	4/14 (28.5%)	0.6570
	Adenomyosis	5/7 (71.5%)	4/14 (28.5%)	0.15

Data are presented as the number of detectable results (n) relative to the total number of measurements (N), together with percentages (%).

**Figure 1:**
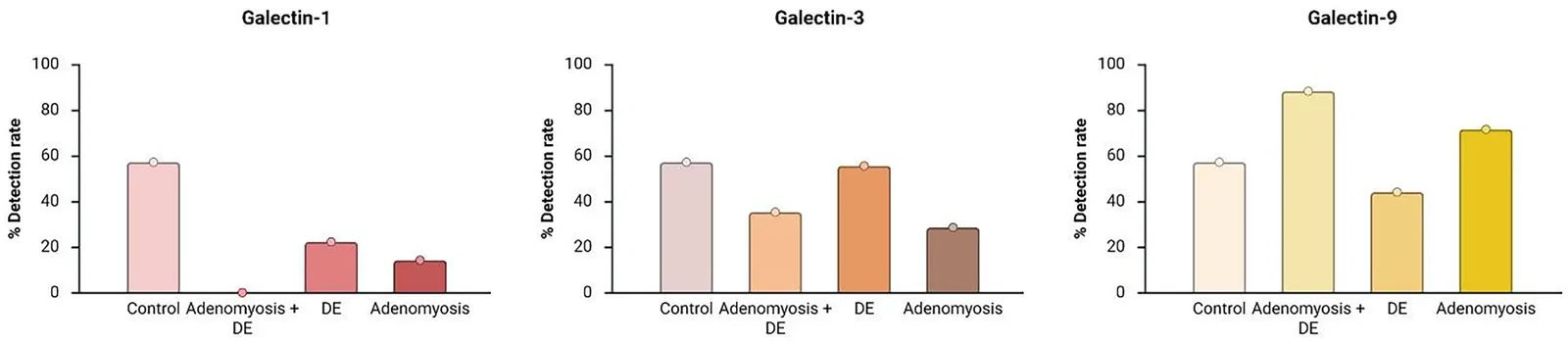
A comparison of detectable Gal-1, Gal-3 and Gal-9 levels between the clinical study groups and the control group.

For galectin-8, all measured values were below the lower limit of quantification and therefore this analyte was excluded from further statistical analysis (**Table 4**).

**Table 4:** Comparison of detectability of Gal-1, Gal-3, and Gal-9 in serum and peritoneal fluid across different disease groups.

Marker	Group	Detectability in serum n/N (%)	Detectability in peritoneal fluid n/N (%)	p (McNemar test)/Bonferroni-adjusted p
Gal-1	Adenomyosis + DE	0/17 (0%)	9/17 (52.9%)	0.0039 */0.0351
	DE	2/9 (22.2%)	2/9 (22.2%)	1
	Adenomyosis	1/7 (14.2%)	3/7 (42.8%)	0.250
Gal-3	Adenomyosis + DE	6/17 (35.2%)	12/17 (70.6%)	0.0313*/0.2817
	DE	5/9 (55.5%)	5/9 (55.5%)	1
	Adenomyosis	2/7 (28.5%)	2/7 (28.5%)	1
Gal-9	Adenomyosis + DE	15/17 (88.2%)	15/17 (88.2%)	1
	DE	4/9 (44%)	9/9(100%)	0.0625
	Adenomyosis	5/7(71.5%)	5/7(71.5%)	1

Data are presented as the number of detectable results relative to the total number of measurements (n/N, %). Statistical analysis was performed using McNemar’s test. p < 0.05.

*Statistically significant result according to McNemar’s test (p < 0.05). The second p-value represents the Bonferroni-adjusted significance level.

Due to the high proportion of values below the lower limit of quantification, galectin-1, -3, and -9 were analyzed as dichotomous variables (detectable vs. non-detectable).

The frequency of detectable Gal-1 levels was significantly lower in patients with coexisting adenomyosis and DE compared with the control group (unadjusted p = 0.0004; Bonferroni-adjusted p = 0.0036). No statistically significant differences were observed in patients with DE alone or adenomyosis alone compared with the control group (**Table 3**). Analysis of Gal-1 detectability showed that, in patients with adenomyosis coexisting with DE, detectability in peritoneal fluid was significantly higher than in serum (unadjusted p = 0.0039; Bonferroni-adjusted p = 0.0351) (**Table 4**).

No statistically significant differences were observed in the frequency of detectable Gal-3 concentrations between the study groups and the control group (p > 0.05) (**Table 3**). In patients with adenomyosis coexisting with DE, Gal-3 detectability in peritoneal fluid was higher than in serum (unadjusted p = 0.0313); however, this difference did not remain statistically significant after Bonferroni correction (adjusted p = 0.2817) (**Table 4**).

The detectability of Gal-9 was significantly higher in the adenomyosis + DE group compared with the control group (unadjusted p = 0.0011; Bonferroni-adjusted p = 0.0099). In the DE and adenomyosis groups, differences compared with the control group did not reach statistical significance (**Table 3**). No statistically significant differences were observed between serum and peritoneal fluid detectability of Gal-9 within the study groups (**Table 4**; **Figure 1**).

### 3.2 Selected immune system mediators

Serum and peritoneal fluid concentrations of asprosin, FSTL1,meteorin, neuregulin-4, ISM1, SFRP2, and TNF-α were measured in patients with deep endometriosis coexisting with adenomyosis, in patients with deep endometriosis, in patients with adenomyosis, and in the control group (**Table 5**).

**Table 5:** Assay sensitivity and non-quantifiable measurements for biomarkers with results below the quantification limit.

Analyte	Manufacturer-reported sensitivity	Quantifiable sample results
Galectin-8	<0.063 ng/mL	None
Meteorin	<0.057 ng/mL	None
Neuregulin-4	<0.056 ng/mL	None
ISM1	<0.064 ng/mL	None
SFRP2	<31 pg/mL	None

Results were obtained for asprosin, FSTL1, and TNF-α, whereas values for the remaining parameters were below the lower limit of quantification (LLOQ), preventing further statistical analyses. For asprosin, FSTL1, and TNF-α, results are presented as medians with interquartile ranges (Q1–Q3) (**Table 6**).

**Table 6:** Comparison of asprosin, FSTL-1, and TNF-α concentrations in serum and peritoneal fluid across the studied clinical groups.

Materials	Material	Group	N	Me	Q1	Q3	p (Kruskal–Wallis test)
Asprosin	Serum	Adenomyosis + DE	17	1.03	0.75	1.13	0.42
		DE	9	1.03	0.91	1.23	
		Adenomyosis	7	1.15	0.90	1.87	
		Control	14	0.83	0.69	1.19	
	Peritoneal fluid	Adenomyosis + DE	17	0.44	0.43	0.52	0.53
		DE	9	0.47	0.47	0.57	
		Adenomyosis	7	0.46	0.45	0.59	
FSTL 1	Serum	Adenomyosis + DE	9	2.74	1.97	2.88	0.17
		DE	5	3.00	2.32	4.25	
		Adenomyosis	6	4.25	3.22	12.2	
		Control	10	4.13	2.54	5.84	
	Peritoneal fluid	Adenomyosis + DE	9	3.91	3.23	7.54	0.053
		DE	5	1.98	1.86	2.43	
		Adenomyosis**	3	11.52	9.25	16	
TNF-α	Serum	Adenomyosis + DE	17	357.5	323.36	800	0.015/0.0900
		DE*	9	800	627.5	800	
		Adenomyosis	7	372.5	242.5	800	
		Control	14	212.5	187.5	232.5	
	Peritoneal fluid	Adenomyosis + DE	17	357.5	282.5	567.5	0.25
		DE	9	277.5	202.5	312.5	
		Adenomyosis	7	447.5	282.5	800	

Results are presented as medians (Me) with interquartile ranges (Q1–Q3). Statistical analysis was performed using the Kruskal–Wallis test. Values at or above the upper limit of detection were assigned a value of 800 pg/mL (maximum measurable concentration of the assay). Values exceeding the upper limit of quantification were assigned the maximum measurable value (800 pg/mL). * p < 0.05; *post-hoc* Dunn’s test showed a significant difference between the DE and control groups (p < 0.001). **The number of observations reflects participants with quantifiable FSTL1 concentrations; measurements below the lower limit of quantification were excluded from quantitative analysis, presented for descriptive purposes only.

Asprosin concentrations did not differ significantly between the study groups in either serum or peritoneal fluid.

For FSTL1, no significant differences between the groups were observed in serum. Similarly, no statistically significant differences were found in peritoneal fluid. However, only three quantifiable FSTL1 measurements were available in the adenomyosis group, and therefore the corresponding summary statistics should be interpreted cautiously and considered descriptive.

Serum TNF-α concentrations differed significantly between the study groups in the unadjusted analysis (unadjusted p = 0.015); however, this difference did not remain statistically significant after Bonferroni correction (Bonferroni-adjusted p = 0.090). A value of 800 pg/mL, corresponding to the upper limit of the reported assay range, was observed in 6 of 17 participants (35.3%) in the adenomyosis + DE group, 5 of 9 participants (55.6%) in the DE group, and 1 of 7 participants (14.3%) in the adenomyosis group. Because values above 800 pg/mL were not independently confirmed, it could not be determined whether these observations represented concentrations exactly at 800 pg/mL or concentrations exceeding the assay range. Dunn’s post-hoc analysis showed significantly higher TNF-α concentrations in the DE group compared with the control group (p < 0.001). No significant differences between the study groups were observed in peritoneal fluid.

Comparison of biomarker concentrations between serum and peritoneal fluid showed significant differences for some of the analyzed proteins. Asprosin concentrations were significantly higher in serum than in peritoneal fluid in the adenomyosis with deep endometriosis group (unadjusted p = 0.0002; Bonferroni-adjusted p = 0.0016). In the DE group, the difference was statistically significant in the unadjusted analysis (unadjusted p = 0.007), but did not remain significant after Bonferroni correction (Bonferroni-adjusted p = 0.056). Similarly, in the adenomyosis group, the difference was significant in the unadjusted analysis (unadjusted p = 0.01), but not after correction (Bonferroni-adjusted p = 0.080).

For FSTL1, concentrations were significantly higher in serum than in peritoneal fluid in the adenomyosis with deep endometriosis group in the unadjusted analysis (unadjusted p = 0.02); however, this difference did not remain statistically significant after Bonferroni correction (Bonferroni-adjusted p = 0.160). The difference was not statistically significant in the DE group (p = 0.144).

For TNF-α, no significant difference between serum and peritoneal fluid was observed in the adenomyosis with deep endometriosis group (p = 0.77) or in the adenomyosis group (p = 0.86). In the DE group, TNF-α concentrations were significantly higher in peritoneal fluid than in serum in the unadjusted analysis (unadjusted p = 0.01); however, this difference did not remain statistically significant after Bonferroni correction (Bonferroni-adjusted p = 0.080), as shown in **Table 7** and **Figure 2**.

**Table 7:** Results of the Wilcoxon test comparing asprosin, FSTL-1, and TNF-α concentrations between serum and peritoneal fluid in the same patients with adenomyosis, deep infiltrating endometriosis (DE), and coexisting adenomyosis and DE.

Marker	Group	N Serum	N Peritoneal fluid	p Wilcoxon test/ Bonferroni-adjusted p
Asprosin	Adenomyosis + DE	17	17	0.0002*/0.0016
	DE	9	9	0.007*/0.056
	Adenomyosis	7	7	0.01*/0.08
FSTL1	Adenomyosis + DE	9	9	0.02*/0.16
	DE	5	5	0.144
TNF-α	Adenomyosis + DE	17	17	0.77
	DE	9	9	0.01*/0.08
	Adenomyosis	7	7	0.86

*Statistically significant result according to Wilcoxon test (p < 0.05). The second p-value represents the Bonferroni-adjusted significance level.

**Figure 2:**
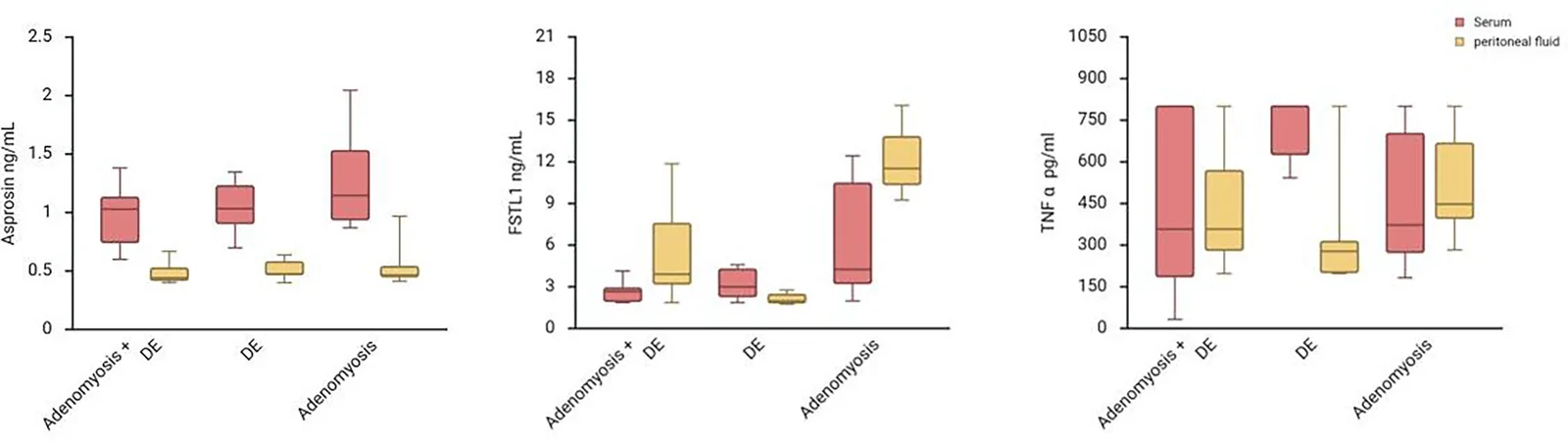
Serum and peritoneal fluid concentrations of asprosin, FSTL-1, and TNF-α in paired samples from patients with adenomyosis, deep endometriosis (DE), and coexisting adenomyosis and DE.

### 3.3 Basic hematological and biochemical parameters

Additionally, all patients underwent assessment of basic laboratory parameters, including hematological indices and selected biochemical parameters. No significant differences were observed between the groups in HGB, PLT, or glucose levels (all p > 0.05) (**Table 8**).

**Table 8:** Selected peripheral blood and biochemical parameters (WBC, white blood cell count; PLT, platelet count; HGB, hemoglobin; CRP, C-reactive protein; GLUCOSE, glucose concentration) in patients with adenomyosis, deep endometriosis (DE), and coexisting adenomyosis and DE.

Marker	Group	N	Me	Q1	Q3
WBC	Adenomyosis + DE	17	6.26	5.33	7.48
	DE	9	6.69	5.13	7.05
	Adenomyosis	7	5.23	4.97	6.86
PLT	Adenomyosis + DE	17	264	232	277
	DE	9	236	223	247
	Adenomyosis	7	255	198	284
HGB	Adenomyosis + DE	17	13.1	12.6	13.5
	DE	9	13.2	12.1	13.7
	Adenomyosis	7	13.1	12.2	13.8
CRP	Adenomyosis + DE	17	0.6	0.6	1.23
	DE	9	1.39	0.75	3.51
	Adenomyosis	7	0.82	0.77	0.84
Glucose	Adenomyosis + DE	17	89,8	85	98.8
	DE	9	90.1	85.3	93.9
	Adenomyosis	7	94.45	90.9	100.7

Data are presented as medians (Me) with lower (Q1) and upper (Q3) quartiles.

In the group of patients with adenomyosis coexisting with deep endometriosis, a moderate positive correlation was observed between asprosin concentration and platelet count (PLT) (r = 0.53). In this same group, no significant associations were found between asprosin and the other hematological parameters.

In the deep endometriosis group, a moderate positive correlation was observed between asprosin concentration and hemoglobin (HGB) (r = 0.50).

Analysis of the relationship between TNF-α and hematological parameters in the adenomyosis coexisting with deep endometriosis group revealed a very weak negative correlation with WBC (r = −0.05) and PLT (r = −0.08) (**Table 9**).

**Table 9:** Spearman correlations between markers (asprosin, TNF-α, FSTL1) and selected hematological parameters in the studied clinical groups.

Parameters	Group	Asprosin			TNF-α			FSTL1		
		Ade+DE	DE	ADE	Ade+DE	DE	ADE	Ade+DE	DE	ADE
WBC	Ade+DE	-0.52			-0.05			0.37		
	DE		0			-0.53			0.4	
	ADE			0.6			-0.77			0.25
PLT	Ade+DE	0.53			-0.08			-0.21		
	DE		-0.03			0.31			-0.3	
	ADE			-0.32			0.52			-0.08
HGB	Ade+DE	-0.20	0.5		0.03			-0.42		
	DE					-0.16			0.7	
	ADE			0.17			0.37			-0.71
CRP	Ade+DE	0.10			0.11			0.08		
	DE		0.1			-0.47			0.1	
	ADE			-0.1			0			-0.5
Glucose	Ade+DE	0.14			-0.43			0.08		
	DE		0.14			-0.41			-0.1	
	ADE			0.37			0.05			-0.82
TNF-α	Ade+DE	-0.02						-0.33		
	DE		0.15						-0.70	
	ADE			-0.41						-0.2
FSTL1	Ade+DE	0.10								
	DE		0.1							
	ADE			-0.37						

Spearman correlation analyses were exploratory and hypothesis-generating. Reported correlation coefficients are unadjusted for multiple comparisons and should not be interpreted as confirmatory evidence. The observed associations require validation in larger, independent cohorts.

## 4 Discussion

The role of galectins in the pathogenesis of endometriosis is currently the subject of numerous studies; however, their significance in disease development has not yet been fully elucidated. Therefore, the present study evaluated the detectability of galectin-1, -3, -8, and -9 in both serum and peritoneal fluid of women with adenomyosis, deep endometriosis (DE), and coexisting adenomyosis with DE. Furthermore, the analysis was expanded to include other immune system mediators, such as asprosin, FSTL1 (follistatin-like protein 1), meteorin, neuregulin-4, isthmin-1 (ISM1), SFRP2, and tumor necrosis factor-α (TNF-α), as well as additional hematological and biochemical parameters. The novelty of the present study lies in the simultaneous assessment of multiple galectins in serum and peritoneal fluid in women with coexisting adenomyosis and deep endometriosis, a phenotype that remains insufficiently characterized in the context of immune-related biomarkers.

In our study, lower detectability of galectin-1 in serum was observed in patients from the adenomyosis groups compared with the control group. This contrasts with the findings of Brubel et al. (8), who reported elevated preoperative Gal-1 levels in women with endometriosis, particularly in advanced stages of the disease (III–IV). The discrepancies may result from differences in the study populations. Importantly, our study assessed galectin levels in terms of detectability due to a high proportion of values below the lower limit of quantification, whereas previous studies typically reported concentration-based measurements. Furthermore, higher detectability of Gal-1 was observed in the peritoneal fluid of patients with adenomyosis + DE compared with serum, indicating a difference in detectability between the two biological compartments. However, this finding does not by itself establish local production of Gal-1 within endometrial lesions or indicate limited diffusion into the systemic circulation. These findings are consistent with previous observations showing overexpression of galectin-1 in endometriotic lesions compared with eutopic endometrium, as well as increased expression in the eutopic endometrium of affected women compared with healthy controls (24). Studies in mouse models have demonstrated that galectin-1 is selectively present in stromal and endothelial cells of endometriotic lesions and is essential for vascular network formation within the lesions. Furthermore, the use of a specific Gal-1 neutralizing antibody resulted in reductions in both lesion size and vascularization. These results highlight the key role of Gal-1 in the local development of endometriotic lesions and suggest that this protein may represent a potential therapeutic target in the treatment of the disease (9).

For galectin-3, higher detectability was observed in the peritoneal fluid compared with serum in patients from the adenomyosis + DE group. This finding may reflect differences in biomarker distribution between the peritoneal and systemic compartments; however, its disease specificity cannot be established because peritoneal fluid samples were not available from the control group. These findings are consistent with previous studies reporting significantly elevated Gal-3 levels in the peritoneal fluid of women with endometriosis, which were also correlated with disease severity and symptom intensity (11). Previous studies have demonstrated significantly elevated expression of galectin-3 in endometrial stromal cells (ESCs) from patients with endometriosis. Moreover, galectin-3 overexpression enhanced the cells’ adhesion and migration capabilities, while reducing NK cell cytotoxicity against ESCs, thereby promoting the survival and implantation of endometrial cells within the peritoneal cavity (10). Studies conducted by Yang et al. (25) demonstrated significantly elevated levels of galectin-3 mRNA and protein in ectopic endometrial tissues compared with both the patients’ eutopic endometrium and control tissues. Further *in vitro* experiments showed that galectin-3 overexpression increased the expression of fibrosis markers, whereas its silencing produced the opposite effect (25).

Similarly, studies in mouse models demonstrated that both the absence of Gal-3 and its inhibition led to a significant reduction in the development of endometrial lesions, decreased lesion size and implantation, as well as reduced expression of VEGF and VEGFR-2, accompanied by lower vascular density and reduced macrophage presence in the tissue (12). The observed higher detectability of galectin-3 in peritoneal fluid may reflect differences in the distribution of this protein between the peritoneal and systemic compartments. However, the present study cannot determine whether this difference results from local production, altered secretion, or differences in protein distribution or clearance. Therefore, peritoneal fluid findings should be interpreted as an association with the local disease environment rather than direct evidence of local galectin-3 production. Additionally, previous studies have suggested that hormonal treatment may modulate Gal-3 expression in endometriotic tissues In women receiving hormonal therapy, decreased Gal-3 expression in ectopic endometrium has been reported compared with controls, together with changes in inflammatory and angiogenic markers, including EMR1, CYP17A, VEGF, and NF-κB, as well as other pathways involved in endometriosis pathophysiology. These findings support the role of hormonal treatment in modulating local inflammatory and tissue-remodeling processes in endometriosis; however, the precise molecular mechanisms underlying these effects require further investigation (26).

In contrast to previous studies reporting significantly elevated serum galectin-3 levels in women with endometriosis compared with controls, we did not observe significant differences in serum Gal-3 detectability between groups. This discrepancy may be attributed to differences in the clinical profiles of the study populations. While the cited study included women with both mild and advanced endometriosis (stages I–IV), our cohort comprised patients with adenomyosis, deep endometriosis, and those with coexisting adenomyosis and deep endometriosis. The heterogeneity of these conditions and concurrent endometrial changes may have influenced serum Gal-3 levels, suggesting that differences in galectin-3 detectability between the peritoneal fluid and serum may warrant further investigation in relation to local and systemic disease processes (27).

In our study, higher detectability of Gal-9 in serum was observed in patients with deep endometriosis coexisting with adenomyosis compared with control serum. These findings are consistent with the observations of Jarollahi et al. (28) who also reported elevated serum Gal-9 levels in women with endometriosis. Previous studies indicated that serum Gal-9 levels were significantly elevated in both mild (stages I–II) and advanced (stages III–IV) endometriosis. In contrast, in our study, no significant differences in serum Gal-9 concentrations were observed between patients with adenomyosis and deep endometriosis and the control group (14). Additionally, other research has shown that in women with endometriosis, Gal-9 detectability were significantly elevated in peripheral blood, whereas in peritoneal fluid, increased TIM-3 expression was observed on CD4+ T cells, along with higher Gal-9 presence across all T and NK cell subsets. In our cohort, Gal-9 detectability was similar in both serum and peritoneal fluid in patients with adenomyosis + DE as well as in women with adenomyosis alone. These results suggest that in the context of more complex endometrial lesions, local and systemic Gal-9 levels may be comparable (29, 30).

In our study, the analysis was extended to include selected immune system mediators, namely asprosin, follistatin-like protein 1 (FSTL1), meteorin, neuregulin-4, isthmin-1 (ISM1), SFRP2, and TNF-α. Quantitative data were obtained only for asprosin, FSTL1, and TNF-α. An interesting avenue for future research is the potential interaction between galectins, asprosin, and FSTL1 via a shared TNF-α–dependent proinflammatory pathway. Such a multiparametric approach could, in the future, aid both in a deeper understanding of molecular mechanisms and in the development of more sensitive diagnostic tools that integrate immune and inflammatory response markers.

Although our observations do not yet allow conclusions regarding direct relationships between galectins, asprosin, and FSTL1, the preliminary results suggest that this combination of parameters represents a promising direction for further research, which should be pursued in larger patient cohorts and using multivariate analyses.

In this study, higher asprosin concentrations were observed in serum compared with peritoneal fluid in patients with adenomyosis, deep endometriosis, and deep endometriosis coexisting with adenomyosis. To date, there are no studies evaluating the role of asprosin in the pathogenesis of endometriosis. Available data on this hormone in women are limited and primarily focus on its potential association with fertility. Studies involving infertile women suggest that asprosin may participate in mechanisms regulating ovarian function, including ovarian reserve (15). This highlights the need for further research, particularly in larger and more homogeneous patient cohorts, to better elucidate the role of asprosin in female reproductive physiology and pathology. Although no studies have directly analyzed the relationship between asprosin and galectins in endometriosis, existing evidence suggests the presence of shared pathophysiological pathways linking these factors. It has been shown that asprosin can enhance inflammatory responses through activation of the NF-κB pathway, leading to increased production of proinflammatory cytokines (31).

Similarly, galectins, particularly Gal-3 and Gal-9, participate in the regulation of the inflammatory response by modulating this signaling pathway (32, 33).

These molecules have been implicated in the regulation of chronic inflammation, which represents a key mechanism in the pathogenesis of endometriosis. Based on published evidence, it can be hypothesized that asprosin, acting primarily at the systemic level, and galectins, exerting local functions within the microenvironment of endometrial lesions, may interact through modulation of the NF-κB axis and its associated inflammatory response. However, this potential interaction was not directly investigated in the present study.

Measurement of FSTL1 levels revealed higher concentrations of this protein in peritoneal fluid compared with serum in patients with adenomyosis + DE. These findings are consistent with studies by Lei et al. (17) which demonstrated significantly increased FSTL1 expression in ectopic endometrial stromal cells (ESCs) and in the peritoneal fluid of women with endometriosis. FSTL1 overexpression in endometrial cells led to upregulation of DIP2A, IL-8, and IL-1β, enhanced cell proliferation, and facilitated macrophage polarization toward the proinflammatory M1 phenotype. These findings suggest that FSTL1 may be associated with inflammatory and angiogenic processes in endometriosis, although the present study does not establish a causal role for FSTL1 in disease development (17). Previous studies have suggested that both FSTL1 and galectins may activate shared proinflammatory pathways, including NF-κB, and may contribute to increased production of cytokines such as TNF-α and IL-1β. However, these mechanisms were not directly investigated or demonstrated in the present study (16, 34–36). In this way, both protein types may mutually reinforce each other, sustaining chronic inflammation, promoting angiogenesis of endometrial lesions, and modulating the immune response within the local microenvironment.

In our study, higher TNF-α levels were observed in the serum of patients with deep endometriosis compared with the control group, and serum concentrations were higher than those in peritoneal fluid. Previous studies evaluating TNF-α levels in the peritoneal fluid and serum of women with endometriosis have produced inconsistent and conflicting results. Some studies report elevated TNF-α concentrations in the peritoneal fluid of patients with endometriosis, particularly in the presence of endometriotic cysts. Endometriosis has also been associated with increased levels of other cytokines involved in Th1- and Th2-mediated immune responses. Furthermore, elevated TNF-α has been shown to enhance the adhesion of endometrial cells to the peritoneum both *in vitro* and *in vivo*. TNF-α has also been investigated as a potential diagnostic marker; however, its clinical utility remains controversial (37–39).

Based on published evidence, an interesting hypothesis is the potential interplay between galectins, asprosin, and FSTL1 through shared TNF-α-dependent proinflammatory pathways. Previous studies have suggested that Gal-1, Gal-3, and Gal-9, as well as asprosin and FSTL1, may influence TNF-α production in stromal cells, macrophages, and lymphocytes, thereby contributing to inflammatory processes, proliferation, and invasion of endometrial cells. However, these interactions were not directly investigated or demonstrated in the present study and therefore should be regarded as a hypothesis for further investigation rather than as an established mechanism (40–44).

Analysis of the relationships between the investigated parameters and hematological indices revealed several noteworthy correlations. In the group of patients with adenomyosis coexisting with deep endometriosis, a moderate positive correlation was observed between serum asprosin levels and platelet count. As this analysis was exploratory and based on a small subgroup, this finding should be considered hypothesis-generating and requires confirmation in larger cohorts. In patients with deep endometriosis, a moderate positive correlation was observed between asprosin levels and hemoglobin; however, given the small subgroup size and exploratory nature of the analysis, the clinical significance of this association remains uncertain and requires further investigation. Conversely, analysis of TNF-α in relation to hematological parameters in the adenomyosis + DE group revealed only very weak negative correlations with WBC and PLT. These findings should also be considered exploratory and require confirmation in larger independent cohorts.

The clinical relevance of adenomyosis and endometriosis extends beyond their local inflammatory and molecular characteristics. Recent clinical evidence has linked adenomyosis with adverse reproductive outcomes, including miscarriage and recurrent pregnancy loss, while endometriosis, particularly deep endometriosis, has been associated with adverse obstetric outcomes such as preterm birth and placenta previa. These observations highlight the broader reproductive and obstetric relevance of the diseases investigated in the present study and provide an important clinical context for further research into their inflammatory and immunological mechanisms. However, the present study was not designed to evaluate reproductive or obstetric outcomes, and therefore no direct conclusions regarding pregnancy outcomes can be drawn from the biomarker findings (45, 46).

In the present study, no quantifiable measurements were obtained for galectin-8, meteorin, neuregulin-4, isthmin-1 (ISM1), or SFRP2 under the assay conditions used. These findings should not be interpreted as evidence of the biological absence of these proteins in serum or peritoneal fluid. Rather, the lack of quantifiable measurements may reflect very low concentrations below the analytical capabilities of the assays and/or differences in assay performance in the analyzed biological matrices. Therefore, the present data do not allow us to distinguish between biological absence and insufficient analytical sensitivity. Further studies using more sensitive and independently validated analytical methods will be necessary to determine whether these biomarkers are present at low concentrations and whether they have potential relevance in endometriosis and adenomyosis.

This study has several limitations that should be acknowledged. First, a formal sample size calculation was not performed, as the study was designed as an exploratory, hypothesis-generating analysis. At the time of study design, limited data were available to estimate expected effect sizes for several of the investigated biomarkers, particularly asprosin, FSTL1, meteorin, ISM1, and SFRP2 in endometriosis. Second, the relatively small sample size reflects the strict inclusion and exclusion criteria applied, as well as the limited availability of well-characterized patients with clearly defined clinical phenotypes in a single-center setting. Although this ensured a homogeneous study population, it also constrained the number of eligible participants. Consequently, the possibility of type II error cannot be excluded, particularly for biomarkers with subtle between-group differences. The statistical power of the paired categorical analyses was also limited by the small subgroup sizes, particularly in the adenomyosis (n = 7) and DE (n = 9) groups. Therefore, non-significant McNemar test results should not be interpreted as evidence of the absence of compartment-specific differences, as the study may have been insufficiently powered to detect modest differences in biomarker detectability between serum and peritoneal fluid. These findings should therefore be considered inconclusive and require confirmation in larger cohorts.

Additionally, the interpretation of some biomarkers was limited by assay detection thresholds, including values below the lower limit of quantification and saturation at the upper detection limit. Five of the eleven targeted biomarkers—galectin-8, meteorin, neuregulin-4, ISM1, and SFRP2—were below the assay-reported LLOQ in all analyzed samples and therefore could not be included in the statistical analysis. For galectin-1, galectin-3, and galectin-9, the high proportion of values below the LLOQ precluded reliable quantitative analysis, requiring the use of binary detectability categories. The reported assay sensitivity and precision were based on the manufacturers’ specifications, and independent analytical validation in serum and peritoneal fluid was not performed. Therefore, it cannot be determined retrospectively whether the low detectability reflected very low biomarker concentrations, matrix-related effects, or limitations of the assay platforms. Importantly, the absence of quantifiable or detectable signals should not be interpreted as evidence that these biomarkers are biologically irrelevant to endometriosis or adenomyosis. Future studies employing more sensitive and/or analytically validated approaches, such as multiplex bead-based assays, electrochemiluminescence immunoassays, or ultrasensitive single-molecule array-based methods, may help to clarify their potential role in these diseases.

An additional limitation concerns the upper quantification limit of the serum TNF-α assay, which was 800 pg/mL. A value of 800 pg/mL was observed in 6 of 17 participants in the adenomyosis + deep endometriosis group, 5 of 9 participants in the deep endometriosis group, and 1 of 7 participants in the adenomyosis group. Because concentrations above 800 pg/mL were not independently confirmed, it is not possible to determine whether these results represented values exactly at the upper limit or concentrations exceeding the assay range. Consequently, the possible influence of upper-limit censoring on the observed distribution and the magnitude of between-group differences cannot be reliably quantified. Therefore, the statistically significant serum TNF-α findings should be interpreted cautiously. Future studies should employ an assay with a higher quantification range and/or an appropriately validated dilution procedure.

Furthermore, peritoneal fluid samples were not available from the control group. Therefore, comparisons between serum and peritoneal fluid could only be performed within the disease groups, which limits the interpretation of differences between these biological compartments and does not allow conclusions regarding disease-specific local production or restricted diffusion into the systemic circulation. The absence of peritoneal fluid samples from the control group also limits the interpretation of biomarker detectability in this compartment. In particular, the higher detectability of Gal-1 and Gal-3 in peritoneal fluid compared with serum in the adenomyosis + DE group cannot be definitively interpreted as a disease-specific feature, because a non-diseased peritoneal fluid reference range was not available. These findings may reflect differences in biomarker distribution between biological compartments; however, they do not establish pathological enrichment, disease-specific local production, or greater clinical relevance of peritoneal fluid compared with serum. Therefore, the potential relevance of the peritoneal compartment should be considered exploratory and requires confirmation in studies including appropriately characterized control participants with available peritoneal fluid samples.

An additional limitation concerns the lack of surgical confirmation of disease-free status in the control group. The control participants did not undergo laparoscopy because there was no clinical indication for an invasive diagnostic procedure. Therefore, the absence of endometriosis or adenomyosis in this group was based on clinical assessment, available imaging findings, and the predefined exclusion criteria rather than histopathological verification. Consequently, undiagnosed mild or asymptomatic disease cannot be completely excluded. If individuals with undiagnosed disease were included in the control group, this potential misclassification could have reduced the contrast between the disease groups and the controls. In particular, it may have attenuated the observed difference in serum TNF-α concentrations between the DE group and the controls. It may also have contributed to the lack of statistically significant differences observed for asprosin and FSTL1, although these findings cannot be attributed solely to possible control-group misclassification. For the remaining biomarkers, the direction and magnitude of the potential bias are uncertain and may depend on the disease phenotype, disease severity, and biological compartment. Because the disease status of the controls was not independently verified and the subgroup sizes were small, the magnitude of this potential bias could not be reliably quantified.

Another limitation concerns the imbalance in BMI and the lack of comprehensive information on the metabolic status of the patients included in the study.

The study groups were not fully balanced with respect to age and BMI. The control group had a higher median age and BMI than the disease groups, which may have introduced additional confounding. This issue is particularly relevant for asprosin, an adipokine associated with adipose tissue mass and metabolic status, and may also be relevant to age-related variation in inflammatory mediators such as TNF-α. Because no multivariable adjustment or age-stratified analysis was performed, the potential independent contribution of age and BMI to the observed biomarker differences cannot be excluded. However, given the small subgroup sizes, particularly in the DE and adenomyosis groups, formal adjustment could have resulted in unstable and unreliable estimates. Therefore, the observed biomarker differences cannot be attributed exclusively to disease status and should be interpreted cautiously. Future studies should include larger, age- and BMI-matched cohorts or apply appropriately powered statistical models to account for these potential confounders.

Another limitation is that the menstrual cycle phase at the time of sample collection was not recorded. Although participants receiving hormonal treatment during the three months preceding sampling were excluded according to the study protocol, the absence of information on menstrual cycle phase prevented us from assessing or adjusting for potential cycle-related variation in biomarker concentrations or detectability. This limitation may be particularly relevant for endocrine- and inflammation-related biomarkers such as TNF-α and galectin-9. Therefore, the observed differences should be interpreted cautiously, as the potential contribution of menstrual cycle phase to the reported biomarker patterns cannot be excluded. Future studies should prospectively document menstrual cycle phase and consider standardizing the timing of sample collection.

Another limitation is that 10 of the 11 investigated biomarkers were measured using assays from the same manufacturer, Cloud-Clone Corp. The assay sensitivity and precision values reported in the present study were based on the manufacturers’ specifications, and independent analytical validation or cross-platform comparison using orthogonal methods was not performed. Consequently, manufacturer- or assay-specific factors, including antibody specificity, matrix effects, and calibration procedures, may have influenced biomarker concentrations or detectability. Although the use of a single manufacturer provided methodological consistency across the analyses, the reproducibility and analytical specificity of the observed biomarker patterns cannot be established from the present study alone. Future investigations should include independent validation and, where feasible, comparison with alternative analytical platforms.

## 5 Conclusion

In summary, the conducted studies demonstrated that in patients with deep infiltrating endometriosis coexisting with adenomyosis, the observed alterations in the detectability of Gal-1, Gal-3, and Gal-9 may indicate the involvement of these parameters in the regulation of immune and inflammatory responses, although the direction and biological significance of these changes require further, more detailed investigations.

Moreover, the observed differences in the concentrations of asprosin, FSTL1, and TNF-α in patients with deep infiltrating endometriosis coexisting with adenomyosis suggest that these parameters may be associated with the inflammatory environment of the disease. However, their biological and clinical significance, as well as potential interactions among these factors, require further investigation in larger and independently validated cohorts.

## Data Availability

The original contributions presented in the study are included in the article/supplementary material. Further inquiries can be directed to the corresponding author.
